# Evaluation of Antioxidant Activity and Growth Control Properties of Nonoscale Structure Produced from *Aloe vera* var. *littoralis* Extract on Clinical Isolates of *Salmonella*

**DOI:** 10.3390/scipharm85030028

**Published:** 2017-07-31

**Authors:** Reza Ranjbar, Mohammad Arjomandzadegan, Hossein Hosseiny

**Affiliations:** 1 Molecular Biology Research Center, Baqiyatallah University of MedicalSciences, 1417613151 Tehran, Iran; ranjbarre@gmail.com; 2 Infectious Diseases Research Center (IDRC) and Department of Microbiology, School of Medicine, Arak University of Medical Sciences, 3813898197 Arak, Iran; 3 Infectious Diseases Research Center, Arak University of Medical Sciences, 3813898197 Arak, Iran; rhhosseiny@yahoo.com

**Keywords:** *Salmonella*, *Aloe vera* var. *littoralis*, antioxidant activity, antimicrobial properties

## Abstract

The aim of the study was to examine antibacterial properties of microemulsion structure produced from *Aloe vera* var. *littoralis* extract as a new tool of nanoscale drug-like materials. *Aloe vera* var. *littoralis* (*A. littoralis*) extract was prepared by distillation method. A nonocarrier structure in the microemulsion system was prepared from the extract. Serial concentrations were prepared from 8 mg/mL extract and the nonocarrier containing 0.1 mg/mL pure extract and were evaluated by a disk diffusion method for 35 *Salmonella* clinical isolates. Minimum inhibitory concentration (MIC) and minimum bactericidal concentration (MBC) were determined by microbroth dilution assay using MTT (3-(4,5-dimethylthiazol-2-yl)-2,5-diphenyltetrazolium bromide) method by an enzyme-linked immunosorbent assay(ELISA) Microplate Reader apparatus. Antioxidant activity of the extract was determined by measuring the ferric reducing ability of plasma (FRAP) assay. From 35 clinical isolates of *Salmonella*, 17 isolates—including resistant isolates of S.E.1103 and S.E.49—had a zone of inhibition (ZI) of 7 to 32 mm in 0.007 mg/mL of the extract. S.E.76 isolate exposed to 30 µg/mL ceftazidime disk had a ZI of 12 mm but had 10 mm in 7µg/mL of *A. littoralis* extract. The inhibitory effect of a nanocarrier at a concentration of 25 µg/mL by 20 mm ZI was comparable by the ceftazidime (30 µg/mL) effect. MIC_50_ was 0.25 mg/mL and MBC_50_ was 0.5 mg/mL by MTT method for the extract. It was shown that *A.littoralis* extract had antioxidant activity of 31.67 µM/mg that could be increased based on concentration. It was concluded that the nanocarrier had a significant effect on the studied isolates in comparison with ordinary antibiotics and had potential for use as a natural antioxidant and antimicrobial material in complementary medicine.

## 1. Introduction

*Aloe vera* can be seen everywhere in human history and has had a persistently influential role in Europe, America, and Asia and other parts of the world [1]. In total, 96% of *Aloe vera* gel is composed of water, but the remaining 4% contains an abundance of ingredients, 75 varieties of which are known. Compounds that have been found in *Aloe vera* gel include polysaccharides, which are able to reduce and heal inflammation. These compounds also have antibacterial and antimicrobial features. Antioxidants such as vitamins A, B, C, E, zinc, amino acids, and essential fatty acids, are also found in these compounds. Calcium is also available in *Aloe vera*; Aloin is a very strong laxative [1,2,3,4,5]. Finding natural antioxidants from plant materials to recreate synthetically can be valuable for healthcare [6].

*Aloe vera* has excellent antitumor and antioxidant properties; it is useful in treatment of wounds and burns, as a blood purifier, analgesic, and glycemic control agent; and it is the best skin moisturizer. Due to these traits, *Aloe vera* is effective for patients with type 2 diabetes, kidney stones, microbial infections, eczema, dysentery, indigestion, and hair loss, among many other issues [1,2,3,4,5].

Salicylic acid and *Aloe vera* containing microemulsion formula (as a skin-soothing agent) without ethyl alcohol was solely reported to contain nanoscale *Aloe vera* gel [7].

Gastroenteritis and diarrheal diseases are considered as the most important health problems worldwide, especially in developing countries [8]. *Salmonella* remains as one of the most important causes of acute gastroenteritis and foodborne infections in many countries such as Iran [9,10,11]. Infections caused by antibiotic resistant *Salmonella* spp. are increasing in Iran and in several previous studies, we have reported high prevalence of multidrug resistant *Salmonella*s pp in this country [12,13,14].

Recently, many herbal medicines with new effects and modern forms have been developed. Ingredients of the plants have been one of the primary routes of treatment in helping to combat diseases in the world. The use of herbs to prevent or cure has a long history and using plant extracts for treatment gained great popularity in the late 1990s [15,16,17].

Many studies were reported on antibacterial properties of *Aloe vera* extracts [18,19,20]. In these studies, *Aloe vera* was used as crude extract (alcoholic, aqueous, etc.).

A nanocarrier can be used as a transport module for other substances, such as drugs. Commonly used nanocarriers include micelles (mono layer), liposomes (multi layers), polymers, and other substances. Nanocarriers are being investigated for their use in drug delivery and their characteristics in chemotherapy.

The aim of the study was to evaluate growth control of *Salmonella* isolates by nanoscale structure produced from *Aloe vera* var. *littoralis* (*A. littoralis*) extract.

## 2. Materials and Methods

### 2.1. Bacterial Isolates

Thirty-five clinical isolates of *Salmonella* were obtained from Research Center of Microbial and Molecular Biology, Baghiyat-Allah University, Tehran, Iran, with defined characteristics [21,22].

### 2.2. Plant Material and Extraction

*A. littoralis* was collected fresh from an herb farm (Isfahan, Iran). Before drying the herbs traditionally, they were washed several times with water and then were kept in a dark place away from sun light at a temperature of around 24 °C for few days to dry and were grounded by a crusher.

### 2.3. Preparation of Extract

Extraction was performed using reflux and distillation. The device was a 1 L balloon equipped by a 40-cm flexuous cooling coil. 200 g of grounded dry *A. littoralis* with 500 mL of water was poured into the reflux apparatus and was stirred for 1 h by a magnetic stirrer without increasing temperature. Temperature was slowly raised to boiling and then was kept constant (100 °C in average). This continued for about 8 h and 100 mL of the extract was obtained. The extract was centrifuged and dried, then kept in sterile containers in a refrigerator.

### 2.4. Nanocarrier Production

In this work, the nanocarrier was prepared in a microemulsion system. Tween-80 (polyoxyethylenesorbitan monooleate, Sigma-Aldrich, Darmstadt, Germany) and Span-20 (sorbitan laurate, Sigma-Aldrich) were blended together with a suitable surfactant at room temperature. The mixture was stirred to obtain a macroscopically homogeneous solution and aqueous extract of *Aloe vera var. littoralis* was added until it turned turbid to determine the maximum solubilization.

This nanoscale structure had a diameter of about 5–10 nm and was a phospholipid-like vesicle containing our extract entrapped in the structure.

### 2.5. The Disk Diffusion Method

Serial concentrations of extract as 8,4, 2, 1, 0.5, 0.25, 0.125, 0.062, 0.031, 0.015,and 0.007 mg/mL were used in a disk diffusion method [21]. In this technique, the isolates were cultured on Mueller-Hinton broth (Himedia, Mumbai, India) and after 24 h incubation at 37 °C a solution containing 1.5 × 10^8^ CFU/mL were prepared from each one. A culture by spread plate method was made on Mueller Hinton agar medium from the suspension by a cotton swab and sterile paper discs were placed on the surface. 20 mL of various concentrations of the extract was poured onto each paper disc; then, they were incubated for 24 h at 37 °C. Diameter of zone inhibition (ZI) was measured using a millimeter ruler. This experiment was repeated three times by the same method. Furthermore, in a Kirby-Bauer Disk Susceptibility Test, ordinary antibiotics were assessed against *Salmonella* isolates including ciprofloxacin (CIP), ampicillin (AM), gentamicin (GM), ceftazidime (CAZ), and tetracycline (TE).

### 2.6. Minimum Inhibitory Concentration Method

The method of Stubbings et al. [23] was used to determine the minimum inhibitory concentration (MIC) of extract against all of studied isolates. Sterile 96-well microplates were used for the assay.

Briefly, a 24-h culture of all clinical isolates was prepared after incubation at 37 °C. The Mueller-Hinton broth containing 1.5 × 10^8^ CFU/mL from each isolate was prepared equivalent to 0.5 McFarland standards and 100 mL of it was added to each well of the 96-bit sterile microplate. Various dilutions were prepared from the extract in each raw by serial dilution; in well numbered 1 to 10 from 4 to 0.007 mg/mL for studied extract and from 0.05 to 0.00018 mg/mL equal to 50 to 18 µg/mL for nanocarrier. Positive control of culture medium containing bacteria was prepared without the extract in well 11 and negative control of pure extract in well 12. Microplates were incubated for 24 h at 37 °C and were examined for turbidity by a BioTek™ ELx800™ Absorbance Microplate Readers (Fisher Scientific, Ottawa, Canada) at 570 nm. The lowest dilution of the extract with any turbidity was considered the MIC [13,24]. All experiments were repeated three times.

Minimum bactericidal concentration (MBC) was determined by the microplate dilution method on the 96-well sterile plate by a complementary adding of MTT (3-(4,5-dimethylthiazol-2-yl)-2,5-diphenyl tetrazolium bromide). The concentration of the extracts which could reduce the viable cell number was determined by MTT method [25,26]. MTT colorimetric assay is based on the capacity of viable cell succinate dehydrogenase enzymes to reduce the water soluble yellow MTT into formazan, an insoluble colored product which is measured by ELISA Microplate Reader apparatus. Since reduction of MTT material could only occur in metabolically active viable cells [6].

Briefly, bacteria treated with extracts for 24 h at 37 °C were incubated with 20 μL of MTT solution for 4 h at 37 °C that was added to each well. In this way, MTT was prepared as 5 mg/mL in phosphate-buffered saline (PBS). Subsequently, the supernatant was slowly removed and 100 μL DMSO (dimethyl sulfoxide) was added to each well.

DMSO is a solvent of formazan crystals and caused varying degrees of intensity of color spectrum of purple to white that is a measure of viable cells. The absorption of wells was measured at wavelength of 570 nm by ELISA reader. In this method, the wells without any absorbance due to formazan crystals were considered to contain only dead cells. The last wells without formazan crystals were considered as MBCs [25,26].

### 2.7. Determination of Antioxidant Properties

The ferric reducing ability of plasma (FRAP) assay was used for the evaluation of the antioxidant power of the samples. In this way, 300 mmol/L acetate buffer, pH 3.6, containing 3.1 g C_2_H_3_NaO_2_· 3H_2_O and 16 mL C_2_H_4_O_2_ per liter of buffer solution, 10 mmol/L TPTZ (2,4,6-tripyridyl-*s*-triazine) in 40 mmol/L HCL; 20 mmol/L FeCl_3_ · 6 H_2_O were used. FRAP reagent was prepared as required by mixing 25 mL acetate buffer, 2.5 mL TPTZ solution, and 2.5 mL FeCl_3_ · 6H_2_O. An aqueous solution of known Fe^2+^ concentration, in the range of 0–1000 μmol/L (FeSO_4_ · 7H_2_O), was used for calibration.

### 2.8. Statistical Analysis

IBM SPSS Statistics12.0 (IBM, Chicago, IL, USA) was used for statistical analyzes. Zones of inhibition from the disc diffusion and absorbance values from microbroth dilution methods were analyzed by SPSS software. The significant difference level was considered at *p*-value < 0.05.

## 3. Results

### 3.1. Bacterial Isolates

Thirty-five clinical isolates from *Salmonella entriditis* (S.E.), *Salmonella typhi* (S.Ty.), *Salmonella flexneri* (S.F.) were obtained from bacterial collection of Molecular Biology Research Center, Baqiyatallah University of Medical Sciences, Tehran, Iran.

### 3.2. Results of Disk Diffusion Method

Studied isolates exposed to serial concentrations of *A. littoralis* extract had various inhibitory effects that are shown in Table 1.

**Figure 1:**
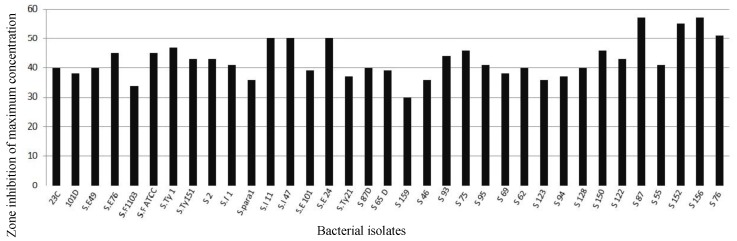
The susceptibility results of *Salmonella* isolates to the serial (maximum) concentrations of *Aloe vera* var. *littoralis* extract .

**Figure 2:**
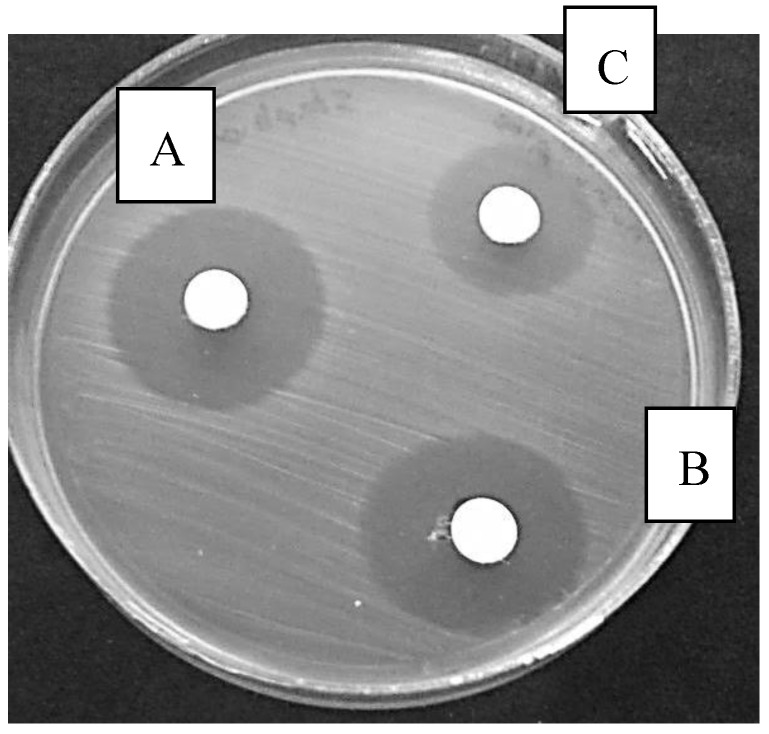
Disk diffusion results for various concentrations of the nanoform of *Aloe vera* extract on *Salmonella* 123. A: 0.125 mg/mL; B: 0.062 mg/mL; C: 0.031 mg/mL.

The extract of *A. littoralis* with the lowest concentration (0.007 mg/mL) had a ZI of 10 mm for isolate S.E.49, 7 mm for S.E.1103, 12 mm for S.E.-ATCC strain, and 25 mm for SI-47 isolate. *A. littoralis* extract in minimum concentration had the greatest effect on isolates number 156 and 152 with 32 and 28 mm, respectively. That means that the extract in a minimum concentration of 0.007 mg/mL had significant effect on the mentioned isolates in comparison with the others that were inhibited at greater concentrations 4 to 0.015 mg/mL (as shown in Figure 1 and Table 1). Isolates numbers 128 and 159 had no ZI at concentration of 0.015 mg/mL. Results indicated that three isolates—156, 152, and 87—formed the largest diameter in all concentrations. Isolate numbers 159 and S.F.1103 had the minimum ZI (Figure 1, Figure 2 and Table 1). Comparison of zones of inhibition in concentrations of 8 and 0.007 mg/mL revealed that the difference was significant (*p* < 0.05).

### 3.3. Antibiotics Sensitivity Testing Results

As the results, isolates numbers of 87D, 101D, 23C, 65D, S.E.49, S.E.ATCC, and S.E.1103 were resistant to antibiotic AM (10 µg) and have been unable to form zone of inhibition. Interestingly, the isolate S.E.76 was susceptible to AM and has a zone diameter of 10 mm, which is equal to the concentration of 0.007 mg/mL (7 µg/mL) of the liquid extract of the *A. littoralis*. That means that the behavior of the clinical isolate S.E.76 was identical when exposing it to chemical and herbal material with identical concentration.

On the other hand, according to the results shown in Table 2, the ZI formed by S.E.76 was 44 mm by antibiotic ciprofloxacin disk containing 5µg drug. That means that CIP was more effective compared to *A. littoralis* extracts when using approximately the same concentration (10 mm in 7 µg/mL) but CAZ was less effective showing 12 mm in 30 µg/mL.

Furthermore, S.E.49, S.E.1103, and S.E.ATCC strains were resistant to some antibiotics but had a significant zone of inhibition at approximately the same extract concentrations.

### 3.4. MIC and MBC Results by Microbroth Dilution Method

**Figure 3:**
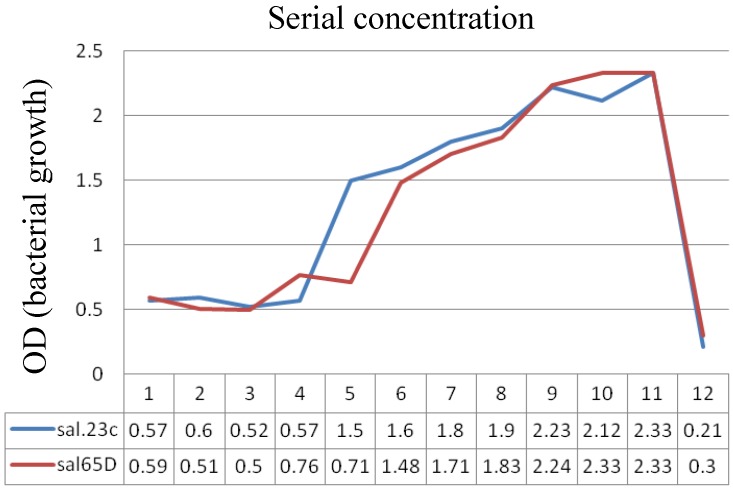
The results of minimum inhibitory concentration(MIC) determined by microdilution method (OD versus concentrations) for two salmonella strains. Concentrations of *A. littoralis* extract in wells 1–12were 4, 2, 1, 0.5, 0.25, 0.125, 0.062, 0.031, 0.015, and 0.007 mg/mL, respectively ( wells 11 and 12 were controls).

The results obtained by the microbroth dilution method and followed by MTT colorimetric assay are shown in Figure 3.

The horizontal line represents the well number from 96-well microplate and the vertical line represents the optical density (OD) and turbidity (equal to bacterial growth). As shown, up to the fourth well, isolate sal23C did not show any growth and the amount of extract in these wells could inhibit the growth of the isolate at MIC (0.5 mg/mL).

The 11th well, which contained bacteria and no extract, showed the greatest amount of turbidity and in the 12th, containing pure extract and no bacteria, the growth was minimum.

When using MTT colorimetric method for the strain 23C, up to the third well, there was no color. The third well had a concentration of 1 mg/mL so this was considered to be MBC. This concentration was lethal for strain 23C in the fourth well.

A similar situation could be observed for sal65D. In this diagram, MIC for sal65D isolate was 0.25 mg/mL. The fifth well, with a concentration of 0.25 mg/mL, is considered as the MIC; growth occurred in the sixth well. MBC for this isolate was 0. 5 mg/mL.

The MIC_50_ was calculated as the median MIC value. Overall, MIC_50_ was calculated for all isolates as 0.25 mg/mL and MBC_50_ was calculated as 0.5 mg/mL by MTT method for *A. littoralis* extract.

### 3.5. Antioxidant Activity

**Figure 4:**
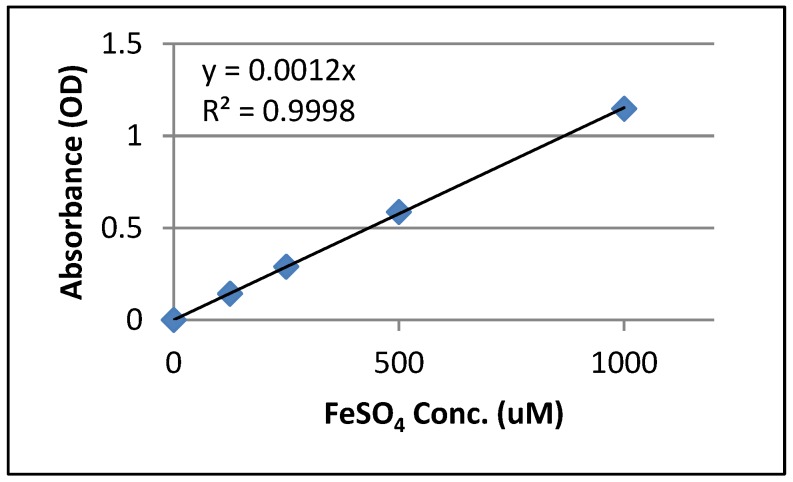
Standard curve for ferric reducing ability of plasma (FRAP) assay.

The FRAP assay provides fast and reproducible results in mixtures of antioxidants in aqueous solution. The dose response characteristics of various concentrations were different, and antioxidant activity decreased with lowering concentration. The antioxidant activity of the *A. littoralis* extract was reduced with decrease in concentration. According to standard curve (Figure 4) and the OD of *A. littoralis* extract, the antioxidant power was 31.67 µM/mg.

## 4. Discussion

Results of the present study showed that aqueous extract of *A. littoralis* could control growth of clinical isolates of *Salmonella* with good antioxidant activities. Interestingly, we have developed a new form of the aqueous extract at the nanoscale that has sufficient antibacterial activity.

These advantages have not been reported earlier. 7. Varanasi and Rariy in 2009 published a US patent on foamable microemulsion from *Aloe vera* only for anti-inflammatory purposes (not antibacterial), such as topical administration [7].

We used microbroth dilution broth and MTT methods for MIC and MBC determination. In many other works, only a disk diffusion method for determining ZI was accomplished [23,24,27,28,29]. According tothe results of the present study, the aqueous extract of *A. littoralis* even in low concentrations could inhibit the growth of clinical strains of *Salmonella*.

It would be proper if the antimicrobial properties would be together with antioxidant activities. For example, antioxidant and antibacterial properties of hawthorn (*Crataegus elbursensis*) seed and pulp extract were investigated by Salmanian S. They reported that the antioxidant activity of the studied extract was not as significantly good as BHT but both pulp and seed extract had inhibitory activity against the four bacteria tested, with the pulp extract showing more activity than the seed extract [6]. In the present work, our nanoscale structure has antioxidant activity.

Asimi et al., in 2013 reported the antioxidant activity and antimicrobial properties of some Indian spices. They mentioned that Cinnamon had the highest antimicrobial effect (12 mm) at maximum concentration on the growth of bacterial strains *Vibrio vulnificus* and *Micrococcus luteus* followed by cumin (9 mm), garlic (8 mm), ginger (8 mm), and turmeric (7 mm) [27]. We determined MIC and MBC of our isolates by microdilution broth and MTT method and MIC_50_ was 0.25 mg/mL and MBC_50_ was 0.5 mg/mL.

In our study, FRAP assay, antioxidant power of the extract had a linear correlation with the concentration of the extract. According to Figure 4, the OD of *A. littoralis* extract was 31.67 µM/mg.

Nejatzadeh worked on aqueous, ethanol, and acetone extract of *Aloe vera* in 2013 and showed that acetone extracts other than aqueous and ethanol extracts had maximum antibacterial activities [28].

Also in 2013, Kedarnath proved that methanol extract of *Aloe vera* had maximum inhibitory effect against *Escherichia coli* but petroleum ether extract had moderate inhibitory effect against *Klebisella*. He compared the ZI (zone of inhibition) of his extracts with gentamicin [29].

In the present study, the ZI was decreased by decreasing concentration of the studied extract. Aqueous extract of *A. littoralis* in the lowest concentration 0.007 mg/mL, had various ZI in S.E.49 and S.E.1103 and S.E.ATCC, S.I.11, S.I.47, S.I.101, and S.E.24 from 7 to 25 mm but no zones were created in the others at the same concentration.

Rishi et al. reported the use of *Aloe vera* phytomodulatory potentials against the inflammation created by OmpR by *Salmonella*. He concluded that *Aloe vera* had a role in the modulation of inflammation modulated by the OmpR systemin *Salmonella* [30].

Waihenya et al., in 2002, worked on infection control of *Salmonella gallinarum* in infected free-range chickens with *Aloe secundiflora*. He indicated that *Aloe secundiflora* crude extract could be used for control of fowl typhoid in chickens. Aloe extract could be created a delay on the clinical signs in studied chickens as well as severity of the disease [31].

Asamenewet al. studied the antimicrobial properties of chromone and anthrone of *Aloeharlana* and found antimicrobial activity against drug resistant *S. typhimurium* with ZI of 0.18 mm [32].

Bisi-Johnson found that African *Aloe vera* and some other medicinal plants had appropriate antibacterial activity against multidrug resistant *Salmonella typhi*, *S. enterica* serotype Isangi, and other isolates as etiological agents of diarrhea. The isolates were sensitive to crude acetone extracts of African *Aloe vera* with MIC ranging 0.018 to 2.5 mg/mL [33].

The above-mentioned reports confirmed our results in antibactericide activities of *A. littoralis* extract. Many antibiotics and chemicals have a bacteriostatic effect. These results, in comparison with antibiogram results, revealed that aqueous extracts of *A. littoralis* have the same effect on these clinical isolates and need to be investigated in vivo.

In the present study, MIC was determined for bacteriostatic and MBC for bactericide properties of the extract. It was proved that MIC_50_ and MBC_50_ of the extract were in low concentrations, which is a good advantage with regard to cost benefits in drug discovery.

## 5. Conclusions

In this study, it was proved that *A. littoralis* could be used as an antimicrobial agent for the control of a variety of clinical species of *Salmonella*. Furthermore, herbal therapy by *A. littoralis* has some benefits—antioxidant properties and a lack of any side effects—in comparison with chemical antibiotics as an appropriate agent in complimentary medicine against salmonellosis.

## Figures and Tables

**Table 1:** Zone of inhibition(ZI) for the clinical strains of *Salmonella* (in mm).

Clinical Isolates	Concentration (mg/mL)										
	8	4	2	1	0.5	0.25	0.125	0.062	0.031	0.015	0.007
23C	41	40	38	35	34	33	32	30	27	19	0
101D	39	38	35	33	31	24	23	14	13	10	0
S.E.49	42	40	37	37	35	34	28	22	20	15	10
S.E.76	46	45	45	40	40	37	35	35	30	15	10
S.F.1103	36	34	30	38	35	27	25	20	15	12	7
S.F.ATCC	47	45	45	38	35	33	27	25	20	17	12
S.Ty.1	49	47	45	43	39	33	32	31	28	25	0
S.Ty.151	44	43	39	37	35	33	30	29	27	26	0
S 2	45	43	40	38	35	33	33	32	30	28	0
S.I 1	43	41	37	33	29	28	23	20	17	13	0
S.para1	37	36	32	32	32	32	31	31	28	25	0
S.I.11	51	50	45	34	32	30	23	20	15	9	9
S.I.47	50	50	45	44	41	39	36	34	33	27	25
S.E.101	40	39	34	34	30	27	24	19	10	10	9
S.E.24	50	50	45	37	34	34	26	21	16	13	7
S.Ty.21	39	37	34	36	33	31	26	24	19	15	10
S 87D	42	40	32	30	30	28	25	20	15	10	0
S 65 D	41	39	37	36	32	26	22	18	12	12	0
159	33	30	27	25	24	20	17	14	11	0	0
46	37	36	28	27	24	23	23	21	21	14	0
93	45	44	40	34	30	30	30	25	19	14	10
75	47	46	38	37	36	30	28	27	19	17	0
95	43	41	38	35	31	30	29	24	19	15	0
69	39	38	32	30	38	26	23	18	15	11	0
62	42	40	36	33	30	28	17	21	17	17	12
123	38	36	34	30	28	24	22	19	17	11	0
94	39	37	32	30	28	26	26	22	17	14	0
128	42	40	36	30	28	25	22	19	15	0	0
150	47	46	38	32	30	29	27	29	23	18	0
122	45	43	34	30	27	26	25	21	18	16	14
87	58	57	53	48	44	41	38	35	32	24	20
55	43	41	40	37	32	25	22	16	15	12	10
152	56	55	52	50	47	44	41	39	37	33	28
156	58	57	54	50	47	44	42	39	34	33	32
76	52	51	49	48	44	44	38	35	32	30	27

**Table 2:** The inhibitory growth zones by the studied isolates exposed to antibiogram discs.

Isolates	Antibiotic (mm)			
	CAZ	CIP	AM	GM
152	18	36	25	27
150	R	40	20	33
221	R	40	20	33
94	R	35	19	32
62	22	38	20	30
156	16	40	23	33
87	R	45	12	34
95	R	35	19	34
75	R	35	20	30
46	R	40	14	28
93	R	35	19	29
123	R	32	20	28
128	R	32	19	30
76	16	31	24	31
S.E.49	R	38	R	18
S.E.76	12	44	10	22
S.E.1103	18	40	R	R
S.E.ATCC	10	R	R	R

CAZ: Ceftazidime (30 µg); CIP: Ciprofloxacin (5 µg); AM: Ampicillin (10 µg); GM: Gentamicin (10 µg); R: Resistant.
